# Physiology and Histology of the Cerebral Convolutions

**Published:** 1879-09

**Authors:** 


PHYSIOLOGY AND HISTOLOGY OF THE CEREBRAL CONVOLUTIONS. ALSO, POISONS OF THE INTELLECT. By Chas. Richet, A.M., M.D., Ph.D. (Former Intern of the Hospital of Paris.) Translated by Edward P. Fowler, M.D. Wm. Wood & Co., N. Y. 1879.

The same editor, presenting this volume, has given a translation of Prof. Charcots work upon Localization in Diseases of the Brain, and the connection between the two is apparent.

The subjects of cerebral physiology and pathology are no easy ones to develope, and the mass of facts that have been adduced, with the various experiments that have been practiced, but reveal the difficulties in the way of the perfect comprehension of the subject.

New discoveries open up new fields of inquiry, and thus the question is extended infinitely. Much has been done, however, in the way of elucidating this complex subject, and the book before us gives a fair exposition of the progress made.

